# Assessing the effects of common variation in the *FOXP2* gene on human brain structure

**DOI:** 10.3389/fnhum.2014.00473

**Published:** 2014-07-01

**Authors:** Martine Hoogman, Tulio Guadalupe, Marcel P. Zwiers, Patricia Klarenbeek, Clyde Francks, Simon E. Fisher

**Affiliations:** ^1^Department of Language and Genetics, Max Planck Institute for PsycholinguisticsNijmegen, Netherlands; ^2^Donders Institute for Brain, Cognition and Behaviour, Radboud University NijmegenNijmegen, Netherlands

**Keywords:** FOXP2, imaging genetics, language, transcription factor, MRI, brain anatomy, VBM

## Abstract

The *FOXP2* transcription factor is one of the most well-known genes to have been implicated in developmental speech and language disorders. Rare mutations disrupting the function of this gene have been described in different families and cases. In a large three-generation family carrying a missense mutation, neuroimaging studies revealed significant effects on brain structure and function, most notably in the inferior frontal gyrus, caudate nucleus, and cerebellum. After the identification of rare disruptive *FOXP2* variants impacting on brain structure, several reports proposed that common variants at this locus may also have detectable effects on the brain, extending beyond disorder into normal phenotypic variation. These neuroimaging genetics studies used groups of between 14 and 96 participants. The current study assessed effects of common *FOXP2* variants on neuroanatomy using voxel-based morphometry (VBM) and volumetric techniques in a sample of >1300 people from the general population. In a first targeted stage we analyzed single nucleotide polymorphisms (SNPs) claimed to have effects in prior smaller studies (rs2253478, rs12533005, rs2396753, rs6980093, rs7784315, rs17137124, rs10230558, rs7782412, rs1456031), beginning with regions proposed in the relevant papers, then assessing impact across the entire brain. In the second gene-wide stage, we tested all common *FOXP2* variation, focusing on volumetry of those regions most strongly implicated from analyses of rare disruptive mutations. Despite using a sample that is more than 10 times that used for prior studies of common *FOXP2* variation, we found no evidence for effects of SNPs on variability in neuroanatomy in the general population. Thus, the impact of this gene on brain structure may be largely limited to extreme cases of rare disruptive alleles. Alternatively, effects of common variants at this gene exist but are too subtle to be detected with standard volumetric techniques.

## Introduction

A significant proportion of children have unexpected problems with acquiring proficient spoken language, despite adequate intelligence and opportunity. Family and twin studies indicate that genetic factors make substantial contributions to the risk of developmental speech and language impairments (Graham and Fisher, 2013). One of the most well-known genes to have been implicated in such disorders is *FOXP2* (Fisher and Scharff, 2009). *FOXP2* encodes a transcription factor, a protein that directly binds to regulatory regions of other target genes and thereby modulates their expression (Vernes et al., 2007).

Disruption of one copy of *FOXP2* leads to problems with mastering coordinated sequences of speech movements (known as childhood apraxia of speech, CAS, or developmental verbal dyspraxia, DVD), accompanied by broad difficulties in language expression and comprehension, also affecting written modalities (Watkins et al., 2002a). A number of distinct etiological mutations affecting this gene have been discovered, in different families and cases (Fisher and Scharff, 2009). These range from missense mutations (Lai et al., 2001; Laffin et al., 2012), non-sense mutations (MacDermot et al., 2005) and indels (Turner et al., 2013), to gross chromosomal abnormalities like translocations (Shriberg et al., 2006; Kosho et al., 2008) and deletions (Zeesman et al., 2006; Palka et al., 2012; Rice et al., 2012; Zilina et al., 2012).

The most thoroughly studied *FOXP2* disruption is a heterozygous missense mutation that co-segregates with speech and language disorder in 15 members of a three generation pedigree, known as the KE family (Fisher et al., 1998). The mutation, which is exclusive to this particular family, yields an arginine-to-histidine substitution in the DNA-binding domain of the encoded protein (Lai et al., 2001), which impairs its function (Vernes et al., 2006). Magnetic resonance imaging (MRI) of the KE family indicates overtly normal brain structure in the affected members, but in-depth statistical analysis using voxel-based morphometry (VBM) has uncovered a number of distributed sites showing significant differences from unaffected people. Bilateral reductions in gray-matter density were noted in the inferior frontal gyrus, caudate nucleus, precentral gyrus, temporal pole, and cerebellum, while increases were reported in the posterior superior temporal gyrus, angular gyrus, and putamen (Watkins et al., 2002b; Belton et al., 2003). Positron Emission Tomography of affected KE subjects on word repetition tasks revealed overactivation of left caudate nucleus, and left premotor cortex, with an extension into Brodmann Area (BA) 44 (Vargha-Khadem et al., 1998). Moreover, in functional magnetic resonance imaging (fMRI) studies with verb-generation tasks, affected family members showed underactivation of the left inferior gyrus and the putamen, even when no vocal output was required (Liégeois et al., 2003). Overall, the inferior frontal gyrus, striatum (in particular the caudate nucleus), and cerebellum are sites of pathology that have been most consistently associated with *FOXP2* disruption in multiple studies (Vargha-Khadem et al., 1998; Watkins et al., 2002b; Belton et al., 2003; Liégeois et al., 2003). Intriguingly, analyses of human brain tissue have shown that deep layers of the cortex, medium spiny neurons of the striatum, and Purkinje cells of the cerebellum, are crucial neuronal subpopulations that most highly express FOXP2 during early development (Lai et al., 2003). These independent findings indicate remarkable overlaps with the neuroimaging findings (Lai et al., 2003).

**Figure 1:**
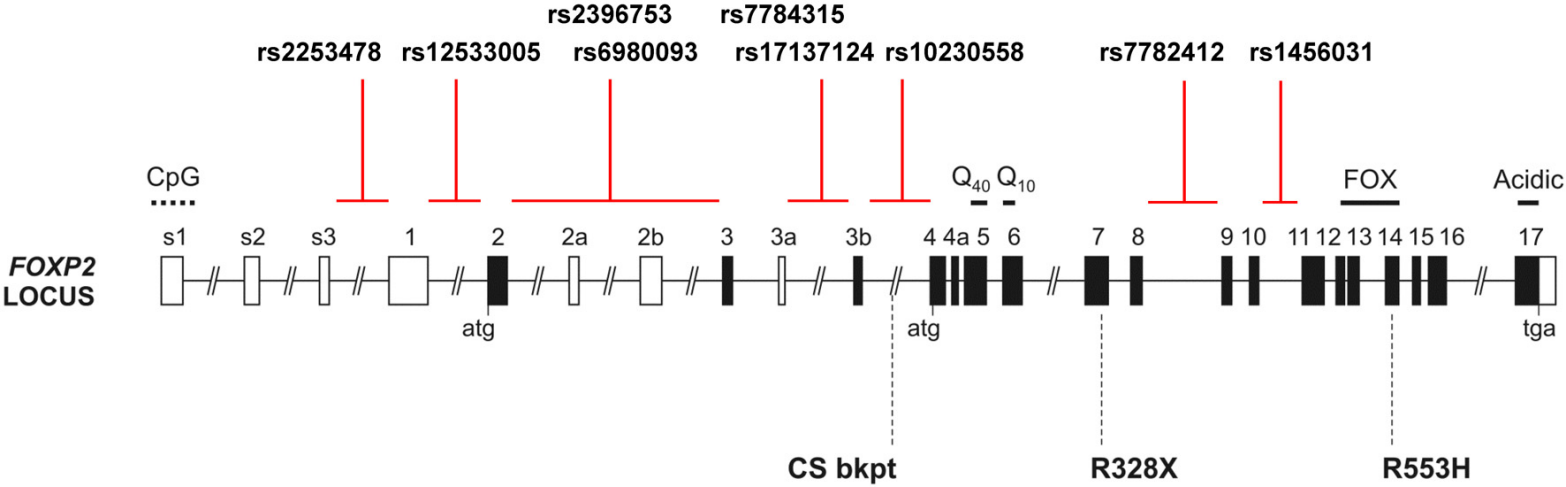
**The human FOXP2 locus**. Schematic of the human *FOXP2* locus, which spans >600 kb in chromosomal band 7q31, showing the intronic locations of candidate SNPs from prior studies of common variation. Black shading indicates translated exons; “atg” and “tga” denote positions of initiation and termination codons. Known domains encoded by exons include polyglutamine tracts (Q40 and Q10), the forkhead domain (FOX), and an acidic C-terminus. Exons 3b and 4a are alternatively spliced coding exons yielding amino acid insertions, whereas alternatively spliced exons 2a, 2b, and 3a are predicted to be non-coding. Exons s1–s3 and 1 represent alternative 5′ UTR regions. CpG marks the site of a CpG island. Three rare disruptive mutations reported in children with severe speech and language impairment are indicated below the locus schematic: the R553H mutation initially discovered in the KE family, an R328X mutation identified in another family and a translocation breakpoint found in an unrelated case (CS) (Lai et al., 2001; MacDermot et al., 2005). Multiple additional point mutations and chromosomal rearrangements have been reported (Graham and Fisher, 2013).

The imaging studies in the KE family have clearly shown that a rare high-penetrant mutation which severely disrupts *FOXP2* is linked with alterations in brain structure and function in the people who carry it, with major consequences for their development of speech and language skills. These intriguing findings have raised new research questions, such as whether or not the same genetic locus harbors common DNA variants with more modest effects on brain structure and function. Do such gene variants have detectable impacts on aspects of brain anatomy, neural activation and/or behavior, in other language-related disorders or in the general population? Researchers have sought to answer these questions by assessing single-nucleotide-polymorphisms (SNPs) in a range of studies with different disorders, and in typically developing people (see Table 1 and Figure 1, for summary).

**Table 1:** **Overview of previous studies of common variation in *FOXP2* and our corresponding regions of interest for VBM analysis**.

**FOXP2 SNP**	**Genomic location (Build 37)**	**Phenotype modality**	**Associated (endo) phenotype**	**Sample size**	**Key references**	**Our region of interest for VBM**
rs2253478	113977996	Behavior	Poverty of speech	293 SZ^a^ patients	Tolosa et al., 2010	Whole brain
rs12533005	114056055	fMRI	Temporo-parietal, inferior frontal activity	19 dyslexics and 14 controls	Wilcke et al., 2012	Parietal, temporal, and inferior frontal lobe
rs2396753	114148331	sMRI	Gray matter dlPFC volume	40 SZ^a^ patients and 36 controls	Španiel et al., 2011	BA9 + BA46
rs6980093	114162740	fMRI	Bilateral inferior frontal activity	94 healthy subjects	Pinel et al., 2012	Inferior frontal lobe
rs7784315	114190643	fMRI	Left precentral activity	94 healthy subjects	Pinel et al., 2012	Left precentral area
rs17137124	114210814	SPECT	Hypoperfusion of frontal and temporal gyrus	96 FTLD^b^ patients	Padovani et al., 2010	Frontal and temporal lobes
rs10230558	114245749	Behavior	Word reading	188 family trios	Peter et al., 2011	Whole brain
rs7782412	114290415	Behavior	Word reading	188 family trios	Peter et al., 2011	Whole brain
rs1456031	114296102	SPECT	Hypoperfusion of various regions in the brain	96 FTLD^b^ patients	Padovani et al., 2010	Frontal, temporal lobe, right putamen, left cingulate gyrus

a SZ, Schizophrenia;

b FTLD, Frontotemporal Lobar Degeneration.

One study assessed four common *FOXP2* SNPs in patients suffering from frontotemporal lobar degeneration (FTLD), a neurodegenerative disorder which can involve breakdown of speech fluency, and reported that alleles of rs1456031 and rs17137124 were additively associated with scores on verbal fluency tasks (Padovani et al., 2010). Based on single-photon emission computed tomography (SPECT) imaging of 96 of the FTLD patients, the authors suggested that alleles of rs1456031 were associated with differential hypoperfusion (local decreased blood flow possibly leading to cell death) in frontal and temporal gyri, right putamen, and left cingulate gyrus. They also reported that rs17137124 variants were associated with differential hypoperfusion in frontal and temporal regions (Padovani et al., 2010). In a subsequent investigation of 34 patients with primary progressive aphasia, the same team proposed that the putative risk alleles of these SNPs were associated with greater hypoperfusion in frontal areas, particularly the left inferior frontal gyrus and the right cingulate gyrus (Premi et al., 2012).

In addition, *FOXP2* polymorphisms have been investigated in relation to schizophrenia, which some researchers propose to be a language-related disorder (Tolosa et al., 2010). A VBM study of neuroanatomy in 40 schizophrenia patients targeted rs2396753, a SNP that had been previously associated with auditory hallucinations (Sanjuán et al., 2006), and reported that the C allele was correlated with reductions in gray matter volume in the dorsolateral prefrontal cortex (dlPFC) (Španiel et al., 2011). Another study in 293 schizophrenia patients suggested that a different SNP, rs2253478, was associated with poverty of speech in schizophrenia, but the relevance of this polymorphism for neuroanatomy was not investigated (Tolosa et al., 2010).

The effects of common variants of *FOXP2* have also been investigated using functional neuroimaging. Wilcke and colleagues assessed rs12533005 in relation to fMRI data from a rhyming task in a cohort of 19 dyslexics and 14 controls, and reported a main effect of the SNP in two temporo-parietal brain areas (the angular and the supramarginal gyrus), as well as an interaction between dyslexia status and SNP alleles, reported to affect activation of inferior frontal regions (Wilcke et al., 2012). Another study, in 94 healthy adults, reported associations of rs6980093 with variations in bilateral inferior frontal activity and rs7784315 with variations in left precentral activity, as assessed by fMRI during a reading task (Pinel et al., 2012). In a behavioral study of 188 family trios with dyslexia, rs10230558, rs12533005, and rs7782412 were associated with articulation and word reading phenotypes (Peter et al., 2011). However, common variation in *FOXP2* remains relatively under-studied with regard to natural variability in language performance in the general population. A recent behavioral study of 456 healthy subjects reported that rs2396753 and rs12533005 were associated with performance on a dichotic listening task; the authors thus proposed that these SNPs modulate hemispheric asymmetries for speech perception, although again there was no neuroimaging data included (Ocklenburg et al., 2013).

As the above literature review shows, the potential impact of common variation in *FOXP2* remains open to debate. In particular, while common *FOXP2* SNPs have been the subject of multiple neuroimaging genetics studies, all such investigations have involved notably small sample sizes with low power and high susceptibility to false positive findings (Button et al., 2013), and there are no reports of independent replications. In the current investigation, we assessed the effects of common variants of *FOXP2* on brain structure using a substantial dataset of 1301 typically developing adult subjects from the general population, a sample which is more than 10 times larger than those used for previous neuroimaging genetic studies of this gene. To provide a statistically robust study design, we carried out our investigation in stages.

First, we checked the common variants of *FOXP2* that have been proposed to have effects on neuroanatomy, function or behavior/cognition in the prior smaller studies (Table 1 and outlined above). Where possible, we tested specific hypotheses regarding particular brain regions, based on the claims made in these previous reports (Table 1). For SNPs that have been argued to affect neuroanatomy, we could focus our analyses on regions highlighted in the relevant earlier study. If a SNP was previously proposed to alter functional activation, we again targeted the site(s) implicated from the prior report, looking in our sample for effects on structure of that candidate region. This strategy is based on well-established findings of convergent functional and structural effects due to rare severe *FOXP2* disruptions; people carrying such mutations show altered activation on language tasks as well as structural changes detectable by volumetric approaches, affecting the same regions (Vargha-Khadem et al., 1998; Watkins et al., 2002b; Liégeois et al., 2003). Moreover, the downstream pathways regulated by FOXP2 include targets that affect both structural and functional properties of neural circuits (Vernes et al., 2011; French et al., 2012). Since we could not make a clear prediction about the expected direction of effect, we carried out statistical tests that were two-tailed. Some of the candidate SNPs had only been assessed in relation to behavior/cognition in prior studies, so in those cases we did not have a predefined brain region of interest (Table 1). Thus, for all candidate SNPs we went on to carry out a broader evaluation of potential effects anywhere in the brain. In the final stage of our investigation, we performed a gene-wide analysis that captured the majority of common variation in *FOXP2*, to systematically assess associations with relevant neuroanatomical phenotypes in our large sample.

## Methods

### Participants

The study sample consisted of healthy adult subjects taking part in the Brain Imaging Genetics (BIG) study in Nijmegen, The Netherlands (Franke et al., 2010). This study was initiated in 2007 and comprises self-reportedly healthy volunteers who participate in studies at the Donders Centre for Cognitive Neuroimaging, Nijmegen, The Netherlands. All subjects have structural MRI data available as part of their involvement in diverse smaller-scale studies and gave their consent to be part of the BIG study. In addition, for 1301 subjects genome-wide genotyping was also available (Guadalupe et al., 2014) and these subjects were selected for the current study. Subjects were of Caucasian descent with no self-reported neurological or psychiatric history, and mainly had a high level of education (80% with a bachelor student level or higher). The median age was 22.0 years (range 18–55 years) and 41% of the sample was male. All participants gave written informed consent and the study was approved by the local ethics committee (CMO Region Arnhem-Nijmegen, The Netherlands).

### Genotyping

To obtain DNA, saliva was collected using Oragene containers (DNA Genotek, Ottawa, ON, Canada). Isolation of DNA was done by the Human Genetics Department of the Radboud University Medical Centre, Nijmegen, The Netherlands. Whole genome genotyping was done using Affymetrix GeneChip SNP, 6.0 (Affymetrix Inc., Santa Clara, CA). For the first stage of analyses, candidate SNPs from prior studies rs2253478, rs12533005, rs2396753, rs6980093, rs7784315, rs17137124, rs10230558, rs7782412, and rs1456031 were extracted from this dataset using PLINK v1.07 (http://pngu.mgh.harvard.edu/purcell/plink) (Purcell et al., 2007). For the second stage of analyses, all SNPs within the gene boundaries of *FOXP2* (USCS Genome Bioinformatics Site, http://genome.ucsc.edu/) including 25 kb flanking regions to capture regulatory sequences, were extracted. SNPs were excluded when they showed a minor allele frequency of less than 1%, failed the Hardy–Weinberg Equilibrium test (*p* < 0.000005) or had a genotyping rate below 95%. This resulted in 1180 SNPs.

### MRI acquisition

Anatomical T1-weighted whole brain MPRAGE scans were acquired at the Donders Centre for Cognitive Neuroimaging using a 1.5T scanner (Sonata and Avanto, Siemens, Erlangen, Germany) or a 3T scanner (Trio and TrioTim, Siemens, Erlangen, Germany). The imaging protocols of the T1 scans included small variations, due to the fact that the images were acquired during several studies. The most common variations included the following parameters and values: TR/TI/TE/sagittal-slices: 2300/1100/3.03/192; 2730/1000/2.95/176; 2250/850/2.95/176; 2250/850/3.93/176; 2250/850/3.68/176; 2300/1100/3.03/192; 2300/1100/2.92/192; 2300/1100/2.96/192; 2300/1100/2.99/192; 1940/1100/3.93/176; and 1960/1100/4.58/176. Slight variations in these imaging parameters have been shown not to affect the reliability of morphometric results (Jovicich et al., 2009).

### MRI processing

To study local differences in gray and white matter related to genetic variation we used a VBM protocol. For this analysis, T1-images were processed using the default procedures of the VBM8 toolbox (http://dbm.neuro.uni-jena.de/vbm/), implemented in SPM8 (http://www.fil.ion.ucl.ac.uk/spm/). Using a unified model, T1-images were bias-field corrected, segmented into gray, white matter, and cerebro-spinal fluid and normalized to standard space (as defined by the Montreal Neurological Institute; MNI) by high-dimensional DARTEL warping (Ashburner, 2007). The resulting images were modulated by the non-linear part of their DARTEL warp field and smoothed with an 10 mm FWHM Gaussian smoothing kernel, providing for an analysis of relative differences in regional gray and white matter volume, corrected for individual brain size.

To study the effects of all common variation in *FOXP2* on candidate brain regions from prior studies of rare variation, volumes of the caudate nucleus, cerebellum and inferior frontal cortex were segmented using FreeSurfer version 5.1 using labels “caudate right” + “caudate left” as caudate nucleus volume; “cerebellum cortex right” + “cerebellum cortex left” as cerebellum volume; and “left and right pars orbitalis” + “left and right pars triangularis” + “left and right pars opercularis” as inferior frontal cortex volume. These volumes were produced with the standard “-recon-all” processing pipeline and default parameters. Estimates of total brain volume (TBV), for inclusion as a covariate, were calculated as the voxel-wise sum of the native gray matter and white matter probability maps from our VBM processing pipeline.

### Stage 1: analyzing candidate SNPs from prior literature

For the VBM analysis the smoothed images were used in multiple regression analysis implemented in SPM8, to test for volumetric differences in relation to SNP genotypes. The effect of each candidate SNP was tested in a separate multiple regression. Genotypes of each SNP were coded to represent a linear allelic additive effect, and age and sex were used as linear covariates. Scanner field strength was also included as a covariate. Gray and white matter analyses were done separately. After grouping according to genotype, outlier analysis implemented in the VBM toolbox identified images with poor quality or artifacts. Images that showed a deviation of more than 1.5 times the interquartile range from the median were excluded from further analysis. To first assess specific claims about SNP associations from the prior literature we applied small volume corrections (*p*_*FWE*_ < 0.05) to the regions proposed by the original report. We used regions defined by the WFU pickatlas and included BA 9+46 as dlPFC, the parietal, temporal and (inferior) frontal lobe, left precentral area, putamen, and cingulate (see Table 1). If in the original VBM study, effects on white matter were reported, then association was only tested in white matter, whereas if the original study posited effects on gray matter, then association was only tested in gray matter. If the original study was an fMRI or SPECT study, we tested both gray and white matter. If a *FOXP2* SNP had been reported to be associated only with a cognitive/behavioral trait (i.e., without investigating a neuroimaging phenotype) in the prior study, then we performed brain-wide analyses using cluster-extent statistics (*p*_*FWE*_ < 0.05) instead of testing for peaks within predefined regions of interest. Clusters were formed using *p*_*uncorrected*_ < 0.001 and corrected for non-stationarity in the data (Hayasaka et al., 2004). For the candidate *FOXP2* SNPs from the prior neuroimaging genetics reports, we also went on to perform a final exploratory search, testing not only in the brain regions of the previous association, but also across the entire brain using a *p*_*uncorrected*_ < 0.001. In such analyses, we split our sample into a 1.5 Tesla discovery cohort (*n* = 648) and a 3 Tesla replication cohort (*n* = 653), a strategy that has been adopted in earlier published investigations of the Nijmegen BIG sample (e.g., Cousijn et al., 2012).

### Stage 2: whole gene analysis

In the second stage of our analyses, we systematically assessed all common variants of *FOXP2* in the BIG dataset. We tested these for effects on volumetric measures of three brain regions, based on prior neuroimaging studies of rare *FOXP2* mutations: the inferior frontal gyrus, caudate nucleus, and cerebellum (Vargha-Khadem et al., 1998; Watkins et al., 2002b; Belton et al., 2003; Liégeois et al., 2003). A whole gene linear regression analysis was performed for each of 1180 SNPs and 3 regional volumes separately using PLINK, and with covariates age, gender, TBV, and field strength of the scanner. A multiple-testing correction was performed by running 10,000 max (T) permutation test using the “mperm” command and saving all the observed and permuted data using the “mperm-save-all” command. These data were combined to create a summed statistic per run for all SNPs at the same time (10,001 in total, one for the observed data and 10,000 for the permuted data). The empirical *p*-value was then estimated by the number of times the sum of the observed summed statistic was smaller than the sum of the permuted statistic, divided by the total number of permutations (10,000) (Bralten et al., 2011). To find out where in the gene the effect was most prominent, the single SNP *p*-values were evaluated. Adding the mperm command in PLINK gives empirical *p*-values for each SNP, corrected for the number of SNPs in the analysis.

## Results

The genotype distributions in our sample are displayed in Table 2. The genotype distribution of rs7784315 resulted in a relatively small group of minor allele homozygotes and therefore we combined this group with the heterozygotes for our association analysis. The imputation quality of rs7782412 was below standard; 39% of the genotype calls had a probability of lower than 0.9, necessitating the use of another SNP as a proxy. The best SNP that could act as a proxy was rs12705966, located 41 kb upstream of rs7782412, and the two markers have an *R*^2^ of 0.66 and a *d*′ of 1.0.

**Table 2:** **Genotype distribution of the study sample (*n* = 1301)**.

**FOXP2 SNP**	**Minor allele**	**Minor allele homozygotes**	**Heterozygotes**	**Major allele homozygotes**	**Total genotyped sample**
rs2253478	A	201 (15.5%)	631 (48.7%)	464 (35.8%)	1296
rs12533005	C	274 (21.1%)	661 (50.8%)	365 (28.1%)	1300
rs2396753	C	228 (18.4%)	604 (48.7%)	409 (33.0%)	1241
rs6980093	G	226 (18.3%)	602 (48.7%)	409 (33.1%)	1237
rs7784315	C	12 (0.9%)	295 (22.7%)	994 (76.4%)	1301
rs17137124	T	279 (22.3%)	629 (50.4%)	341 (27.3%)	1249
rs10230558	T	286 (22.0%)	657 (50.5%)	358 (27.5%)	1301
rs12705966^a^	G	166 (12.7%)	576 (44.3%)	559 (43.0%)	1301
rs1456031	C	370 (28.4%)	653 (50.2%)	278 (21.4%)	1301

a This was used as a proxy for rs7782412 as described in the Results section.

### VBM results

An effect of rs2396753 on gray matter volume in the dlPFC that was suggested in a previous VBM study (Španiel et al., 2011) could not be replicated in the current study. There were no significant voxels at *p*_*uncorrected*_ = 0.001, nor were there any other effects elsewhere in the brain.

**Figure 2:**
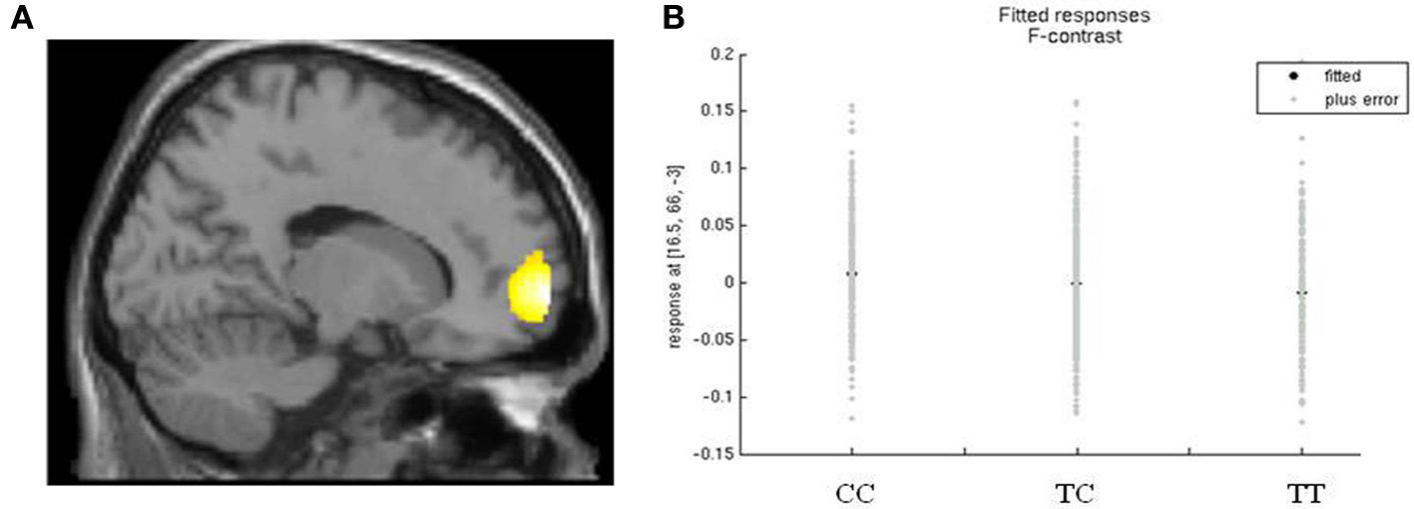
**rs17137124 and white matter density in the frontal lobe. (A)** Relationship between white matter volume in the frontal lobe and variation in rs17137124, at *x* = 16. **(B)** Direction of the effect of rs17137124 at peak voxel *x* = 16, *y* = 66, *z* = −3, with the CC group having the highest white matter values. Voxel co-ordinates are given in MNI (Montreal Neurological Institute) space.

Variation in rs17137124, previously associated with frontal degeneration (Padovani et al., 2010), showed a weak association with white matter in the frontal lobe, *F* = 18.82, *p*_*FWE*_ = 0.04, peak voxel at 16, 66, −3, cluster size = 1263, Figure 2. White matter density was highest in the group of C-allele homozygotes.

The other proposed candidate SNPs listed in Table 1 (Padovani et al., 2010; Tolosa et al., 2010; Peter et al., 2011; Pinel et al., 2012; Wilcke et al., 2012) did not show any association with the structural brain phenotypes measured with VBM either using a region of interest or in a brain wide analysis.

### Whole gene analysis

The results of the gene-wide analysis revealed no significant whole gene *FOXP2* effects on caudate nucleus (*p*_*empirical*_ = 0.81), cerebellar gray matter (*p*_*empirical*_ = 0.71) or inferior frontal volume (*p*_*empirical*_ = 0.84). Some SNPs were suggestive (uncorrected *p*-value < 0.05) of an association with the caudate nucleus volume. However, these results did not survive correction for multiple testing (Table 3). The SNP rs144807019 had the lowest *p*-value (*p*_*uncorrected*_ = 0.002, *p*_*corrected*_ = 0.16). This SNP is in close proximity (~4 kb) to rs1456031, a SNP previously associated with poverty of speech (Tolosa et al., 2010).

**Table 3:** **Suggestive *FOXP2* SNPs for the association with caudate nucleus volume**.

**FOXP2 SNP**	**Genomic location location (Build 37)**	**Uncorrected *p*-value**	**Corrected *p*-value**
rs144807019	114299758	0.001998	0.1588
rs17508329	113799165	0.008991	0.5594
rs7787510	114291436	0.01698	0.8971
rs17213159	114298708	0.01798	0.9081
7:114236165:I	114236165	0.01898	0.8721
rs77390484	113834790	0.02498	0.969
rs2894712	114255168	0.02498	0.97
rs59676751	114213751	0.02597	0.969
rs12671286	114214354	0.02597	0.969
rs12671308	114214491	0.02597	0.969
rs113570114	113865771	0.03097	0.99
7:114236162:I	114236162	0.03097	0.984
rs6951781	114279035	0.03397	0.992
rs6966051	114296104	0.03397	0.985
7:113989183:I	113989183	0.03497	0.994
rs60540213	114202461	0.03696	0.995
rs7788346	114213243	0.03696	0.995
rs7780785	114216749	0.03696	0.995
rs35662342	114217735	0.03696	0.995
rs35335680	114217910	0.03696	0.995
7:114218018:D	114218018	0.03696	0.995
rs1378765	114227026	0.03696	0.995
rs12669360	114228805	0.03696	0.995
rs957523	114349656	0.03696	0.994
rs143156746	113755078	0.04196	0.99
rs6946881	114311726	0.04196	0.998
rs1378769	114355097	0.04196	0.995
rs7806844	114358561	0.04196	0.995
rs2894697	114026090	0.04396	0.994
rs58552483	114147781	0.04396	0.998
rs73436158	114148169	0.04396	0.998
rs35325998	114176415	0.04396	0.998
rs12674004	114176851	0.04396	0.998
rs35487108	114179030	0.04396	0.998
rs73429347	114183387	0.04396	0.998
rs189203299	114190450	0.04496	0.998

## Discussion

The present study went beyond the established impact of rare highly penetrant mutations of *FOXP2* on brain structure, to investigate whether frequent polymorphisms at this locus have effects on normal variation in neuroanatomy in the general population. In the first stage, we targeted common SNPs that have been claimed to have phenotypic effects in prior studies. Despite using a large sample of 1301 healthy participants, more than 10 times larger than any prior neuroimaging genetics study of *FOXP2*, we did not detect significant associations. The sole exception was rs17137124, showing a borderline significant association with white matter density in the frontal lobe, an effect that would not be robust to adjustment for multiple testing.

The lack of effects may be explained by several different factors, which are not mutually exclusive. First, we note that the previous positive findings in neuroimaging genetics of common variations in *FOXP2* have all come from studies with small sample sizes, in scanned groups ranging from a maximum of 96 (Padovani et al., 2010) to as few as 14 participants (Wilcke et al., 2012). Small sample sizes in imaging genetics studies not only lead to reduced power, they make the analyses susceptible to an elevated rate of type I errors (Button et al., 2013). Therefore, at least some of the original reports of SNP associations may represent false-positive findings, especially since the *p*-values in many of these studies were only marginally significant. Second, some of the prior studies of common *FOXP2* variants involved analyses of task-related activations via functional neuroimaging or associations with behavioral traits, whereas the current study focused on effects on brain structure. Thus, it is conceivable that the candidate SNPs are associated with alterations in aspects of brain function or behavioral output without detectable impacts on neuroanatomy. On the other hand, as discussed further below, prior studies of *FOXP2* disruptions have demonstrated effects on both function and structure of the relevant brain circuits (Vargha-Khadem et al., 1998; Watkins et al., 2002b; Liégeois et al., 2003), and the gene is known to regulate targets with roles in neurite outgrowth, axon guidance, and synaptogenesis (Vernes et al., 2011). Future genetic association studies involving functional neuroimaging during language-related tasks in large samples (hundreds, rather than tens of individuals) are needed to properly address this issue. Third, a number of the previous investigations targeted disease cohorts (FTLD, schizophrenia or dyslexia) while this study involved an unselected general population sample. Thus, it might be argued that effects of some of these variants are only relevant for modulating phenotypes within people who have a disorder. However, these are all common disorders, and given the high frequency of the relevant SNPs in healthy individuals, one might expect to uncover some evidence of association with a relevant endophenotype in a large sample such as that used here. Moreover, while *FOXP2* is itself poorly investigated in relation to language skills in the normal range, it has been shown that targets downstream of this transcription factor have effects that are not only relevant to disorder but also to language performance in the general population (Vernes et al., 2008; Whitehouse et al., 2011).

As far as we are aware, for the candidate SNPs of *FOXP2* that have been claimed to have effects on brain structure or function in prior studies, no empirical studies have been carried out to determine their likely impact at the molecular or cellular level. None of the known candidate SNPs change the amino-acid sequence of the encoded protein, so they do not affect its shape or its functional properties (Figure 1). DNA variants that do not alter protein sequences can still have effects on function, for example, by altering how much of the relevant protein is made in any particular cell, how the protein levels are able to change in respond to signals, and/or another aspect of its regulation (Fisher, 2006). The effects of such common regulatory SNPs are typically subtle and can be difficult to demonstrate. Moreover, when multiple common SNPs lie close to each other and tend to be coinherited (i.e., they are in linkage disequilibrium) it is hard to determine which of the neighboring variants provides the functional explanation for an observed association with a phenotypic trait. So far, studies of common *FOXP2* variation have simply assumed that the associated SNPs must be regulatory variants (or are in linkage disequilibrium with regulatory variants) that modulate the expression of the gene, in some undetermined way, without testing this assumption in a cellular assay or other model system. The lack of experimental studies on common variants, in cellular and animal models, is in stark contrast to the in-depth work that has been performed for rare mutations of this gene (Vernes et al., 2006, 2011; Groszer et al., 2008; French et al., 2012; Kurt et al., 2012). It will be important in future to use functional genomics in model systems to increase our understanding of how non-coding regulatory sequences at the *FOXP2* locus affect its expression and function. Findings from such efforts should be closely integrated with ongoing work on phenotypic associations in human datasets, whether from disease cohorts or healthy populations, to increase chance of uncovering biologically valid results (Deriziotis and Fisher, 2013).

A fully gene-wide view did not uncover any common *FOXP2* SNPs as new candidates for having effects on brain structure, at least for the neuroanatomical phenotypes that we were able to study here. Our focus here was on brain structures that have been robustly connected with *FOXP2* functions in prior work on rare mutations and animal models. It is well-established that disruption of the *FOXP2* gene yields detectable alterations of distributed corticostriatal and corticocerebellar brain circuits, affecting both their structural architecture and functional properties. This conclusion is supported not only by neuroimaging studies of humans carrying heterozygous mutations that disturb FOXP2 protein function (as described in the Introduction), but also by diverse investigations of genetically manipulated animal models (Fisher and Scharff, 2009). For example, for mice that carry *Foxp2* mutations, matching those implicated in speech and language disorder, there have been reports of effects on neurite outgrowth (Vernes et al., 2011), task-related neural firing (French et al., 2012), and synaptic plasticity (Groszer et al., 2008) in the relevant brain regions, associated with deficits in acquisition of motor-skills and impaired learning of auditory-motor associations (Kurt et al., 2012). Knockdown of the avian ortholog in a key striatal nucleus of the zebrafinch brain reduces spine density (Schulz et al., 2010), disturbs dopaminergic modulation of corticostriatal signaling (Murugan et al., 2013), leading to reduced vocal plasticity and impaired learning of song (Haesler et al., 2007). Thus, the choice of brain structures for the current gene-wide study of common variants was strongly grounded in existing knowledge about the roles of the gene, but no evidence of effects on the structures of relevant regions could be detected.

Given our sample size and design, we estimated our candidate region VBM analyses had sufficient power (80%) to detect allelic effects small enough to explain 1.7% of the phenotypic variance voxel-wise. Our exploratory VBM analyses had enough power to detect effects as small as 3.4% of the voxel-wise variance, while our gene-wide analysis of *FOXP2* variants had enough power to detect an effect of 2% on the phenotypic variance in regional volumes (calculated in G^*^Power; Faul et al., 2007). It is thus possible that common variations at the *FOXP2* locus do not contribute to variability in relevant aspects of neuroanatomy in the general population, and that its effects on brain structure are mainly evident in extreme cases of rare disruptive alleles. Alternatively, there might be common variants at this gene with effects of a subtle nature, or which impact on aspects of neuroanatomy that are more difficult to detect with standard volumetric techniques. In particular, given the prior evidence of a link between *FOXP2* and neurite outgrowth and axon guidance, investigations of structural and functional connectivity and common SNPs in sufficiently large samples may prove informative.

### Conflict of interest statement

The authors declare that the research was conducted in the absence of any commercial or financial relationships that could be construed as a potential conflict of interest.
